# Dietary meat and fat intake and prevalence of rhinoconjunctivitis in pregnant Japanese women: baseline data from the Kyushu Okinawa Maternal and Child Health Study

**DOI:** 10.1186/1475-2891-11-19

**Published:** 2012-03-27

**Authors:** Yoshihiro Miyake, Keiko Tanaka, Hitomi Okubo, Satoshi Sasaki, Masashi Arakawa

**Affiliations:** 1 Department of Preventive Medicine and Public Health, Faculty of Medicine, Fukuoka University, Fukuoka, Japan; 2 Department of Social and Preventive Epidemiology, Graduate School of Medicine, The University of Tokyo, Tokyo, Japan; 3 Course of Wellness, Graduate School of Tourism Sciences, University of the Ryukyus, Okinawa, Japan

## Abstract

**Background:**

Dietary fat exerts numerous complex effects on proinflammatory and immunologic pathways. Several epidemiological studies have examined the relationships between intake of fatty acids and/or foods high in fat and allergic rhinitis, but have provided conflicting findings. The current cross-sectional study investigated such relationships in Japan.

**Methods:**

Study subjects were 1745 pregnant women. The definition of rhinoconjunctivitis was based on criteria from the International Study of Asthma and Allergies in Childhood. Information on dietary factors was collected using a validated self-administered diet history questionnaire. Adjustment was made for age; gestation; region of residence; number of older siblings; number of children; smoking; secondhand smoke exposure at home and at work; family history of asthma, atopic eczema, and allergic rhinitis; household income; education; and body mass index.

**Results:**

The prevalence of rhinoconjunctivitis in the past 12 months was 25.9%. Higher meat intake was significantly associated with an increased prevalence of rhinoconjunctivitis: the adjusted odds ratio between extreme quartiles was 1.71 (95% confidence interval: 1.25-2.35, *P *for trend = 0.002). No measurable association was found between fish intake and rhinoconjunctivitis. Intake of total fat, saturated fatty acids, monounsaturated fatty acids, n-3 polyunsaturated fatty acids, α-linolenic acid, eicosapentaenoic acid, docosahexaenoic acid, n-6 polyunsaturated fatty acids, linoleic acid, arachidonic acid, and cholesterol and the ratio of n-3 to n-6 polyunsaturated fatty acid intake were not evidently related to the prevalence of rhinoconjunctivitis.

**Conclusions:**

The current results suggest that meat intake may be positively associated with the prevalence of rhinoconjunctivitis in young adult Japanese women.

## Background

Allergic rhinitis is one of the most common disorders in Japan. In a study of 1540 Japanese aged 20 to 49 years, the prevalence of allergic rhinitis was 44.2%, and 89.6% of the subjects with allergic rhinitis were sensitized to Japanese cedar pollen [1]. The prevalence of Japanese cedar pollinosis increased 2.6-fold between 1980 and 2000 [2]. Such an increase may be a consequence of dietary change: a study of Japanese men showed an increase in intake of fat from 5% to 20%, of fish from 56 g/day to 71 g/day, and of meat from 13 g/day to 92 g/day from 1958 to 1999 [3].

Dietary fat exerts numerous complex effects on proinflammatory and immunologic pathways [4]. Several epidemiological studies have examined the relationships between intake of fatty acids and/or foods high in fat and allergic rhinitis in children [5-12] and adults [13-18], but have provided conflicting findings. Such epidemiological evidence in Japan, where fish intake is high, is scarce. A significant inverse relationship was observed between arachidonic acid intake and the prevalence of rhinoconjunctivitis in schoolchildren aged 6-15 years [11], while the sum of intake of eicosapentaenoic and docosahexaenoic acids was significantly associated with a lower prevalence of allergic rhinitis in pregnant women [16]. Another cross-sectional study of female university students showed a significant inverse association between seafood intake and pollen allergy [17].

To accumulate further epidemiological evidence, we performed a cross-sectional study to investigate the relationship between consumption of meat, fish, and specific types of fatty acids and the prevalence of rhinoconjunctivitis in pregnant Japanese women using baseline data from the Kyushu Okinawa Maternal and Child Health Study (KOMCHS).

## Methods

### Study population

The KOMCHS is an ongoing prospective prebirth cohort study that investigates risk and preventive factors for maternal and child health problems such as allergic disorders. From April 2007 to March 2008, the KOMCHS requested that 131 obstetric hospitals in Fukuoka Prefecture, the largest prefecture on Kyushu Island in southern Japan with a total population of approximately 5.04 million, provide as many pregnant women as possible with a set of leaflets explaining the KOMCHS, an application form to participate in the study, and a self-addressed and stamped return envelope. From May 2007 to March 2008, the KOMCHS also requested that forty obstetric hospitals in Okinawa Prefecture, an island in the southernmost area in Japan with a total population of nearly 1.37 million, provide pregnant women with a similar set of materials. In addition, to increase the sample size, from August 2007 to March 2008, pregnant women living in six prefectures on Kyushu Island (other than Fukuoka Prefecture), with a total population of approximately 8.22 million, were provided with a similar set of materials at 252 obstetric hospitals. Pregnant women who intended to participate in the KOMCHS returned the application form to the data management center. When all responses were tallied, a total of 1757 pregnant women between the 5th and 39th weeks of pregnancy had provided their fully informed written consent to participate and had completed the baseline survey. Incomplete data on the variables under study caused the exclusion of 12 pregnant women; in the end, data on 1745 pregnant women were available for analysis. The ethics committee of the Faculty of Medicine, Fukuoka University approved the KOMCHS.

### Measurements

In the baseline survey, each participant filled out a set of two self-administered questionnaires and mailed them to the data management center. Research technicians clarified missing or illogical data by telephone interview.

A self-administered, semi-quantitative, comprehensive diet history questionnaire (DHQ) was used to assess dietary habits during the preceding month [19,20]. Estimates of daily intake of foods (total of 150 foods), energy, and selected nutrients were calculated using an ad hoc computer algorithm for the DHQ based on the Standard Tables of Food Composition in Japan [21]. Information on dietary supplements was not used in the calculation of dietary intake. Detailed descriptions of the methods used for calculating dietary intake and the validity of the DHQ were published elsewhere [19,20]. The correlation coefficients for nutrient intake between those estimated from the DHQ and those observed by a three-day dietary record were 0.75, 0.50, 0.37, and 0.49 for saturated fatty acids, monounsaturated fatty acids, polyunsaturated fatty acids, and cholesterol, respectively, in women [19]. A highly positive correlation was also observed between marine-origin n-3 polyunsaturated fatty acid intake estimated by the DHQ and the corresponding concentration in the serum phospholipid fraction in women (*r *= 0.69) [20]. Energy-adjusted intake by the residual method was used for the analyses [22]. Body weight and height were self-reported as part of the DHQ. Body mass index was calculated as weight (kg) divided by the square of height (m).

A second self-administered questionnaire included questions on rhinoconjunctivitis based on the International Study of Asthma and Allergies in Childhood (ISAAC) [23]. The presence of rhinoconjunctivitis was defined as a positive response to the following two questions: 'In the last 12 months, have you had a problem with sneezing or a runny or blocked nose when you did not have a cold or flu?' and 'In the last 12 months, has this nose problem been accompanied by itchy-watery eyes?' The questionnaire also elicited information on age; gestation; region of residence; number of older siblings; number of children; smoking habits; secondhand smoke exposure at home and at work; family history of asthma, atopic eczema, and allergic rhinitis; household income; and education. A family history of asthma, atopic eczema, or allergic rhinitis (including Japanese cedar pollinosis) was considered to be present if one or more parents or siblings of the study subjects had been diagnosed by a physician as having any of these allergic disorders.

### Statistical analysis

Intake of the dietary factors under study was divided into quartile ranges based on distribution among all study subjects. Age; gestation; region of residence; number of older siblings; number of children; smoking; secondhand smoke exposure at home and at work; family history of asthma, atopic eczema, and allergic rhinitis; household income; education; and body mass index were selected *a priori *as potential confounding factors. Because values of dietary intake under study were energy-adjusted according to the residual method, estimated energy from protein and carbohydrates and alcohol intake were not included in the multivariate model.

Logistic regression analysis was performed to estimate crude odds ratios (ORs) and 95% confidence intervals (CIs) of rhinoconjunctivitis according to the quartile of dietary factors under study, with the lowest quartile as the reference. Multiple logistic regression analysis was used to adjust for potential confounding factors. Trend of association was assessed by a logistic regression model assigning consecutive integers (1 to 4) to the quartiles of the exposure variables. All statistical analyses were carried out using the SAS software package version 9.2 (SAS Institute, Inc., Cary, NC, USA).

## Results

The prevalence of rhinoconjunctivitis in the past 12 months was 25.9% among the 1745 pregnant women. The mean age of the participants and gestation at baseline were 31.2 years and 18.5 weeks, respectively (Table 1). Many more women had a family history of allergic rhinitis than a family history of asthma or atopic eczema. Mean daily total energy consumption, mean daily energy-adjusted intake of meat, and mean daily energy-adjusted intake of total fat during pregnancy were 7434.2 kJ, 64.4 g, and 58.0 g, respectively (Table 2).

**Table 1:** Distribution of selected characteristics in 1745 pregnant women, Kyushu Okinawa Maternal and Child Health Study, Japan

Variable	*n (%) *
Age, years, mean ± SD	31.2 ± 4.3
Gestation, weeks, mean ± SD	18.5 ± 5.4
Region of residence	
Fukuoka Prefecture	971 (55.6)
Other than Fukuoka Prefecture in Kyushu	592 (33.9)
Okinawa Prefecture	182 (10.4)
Having one or more older siblings	914 (52.4)
Having one or more children	1042 (59.7)
Having ever smoked	563 (32.3)
Ever experiencing secondhand smoke exposure at home	1315 (75.4)
Ever experiencing secondhand smoke exposure at work	1106 (63.4)
Family history of asthma	330 (18.9)
Family history of atopic eczema	303 (17.4)
Family history of allergic rhinitis	759 (43.5)
Household income, yen/year	
< 4,000,000	632 (36.2)
4,000,000-5,999,999	618 (35.4)
≥ 6,000,000	495 (28.4)
Education, years	
< 13	428 (24.5)
13-14	577 (33.1)
≥ 15	740 (42.4)
Body mass index, kg/m^2^, mean ± SD	21.4 ± 2.8

**Table 2:** Distribution of daily intake in 1745 pregnant women, Kyushu Okinawa Maternal and Child Health Study, Japan*

Variable	Mean (SD)
Total energy, kJ	7434.2 (2057.0)
Meat, g	64.4 (28.8)
Fish, g	46.7 (25.8)
Total fat, g	58.0 (11.5)
Saturated fatty acids, g	16.8 (4.3)
Monounsaturated fatty acids, g	20.3 (4.9)
n-3 Polyunsaturated fatty acids, g	2.3 (0.7)
α-Linolenic acid, g	1.7 (0.5)
Eicosapentaenoic acid, g	0.17 (0.12)
Docosahexaenoic acid, g	0.29 (0.17)
n-6 Polyunsaturated fatty acids, g	11.0 (2.5)
Linoleic acid, g	10.7 (2.4)
Arachidonic acid, g	0.13 (0.04)
Cholesterol, mg	284.6 (96.4)

* Nutrient and food intake were adjusted for total energy intake using the residual method

Table 3 presents ORs and 95% CIs for the prevalence of rhinoconjunctivitis by quartiles of dietary intake of meat and fish. Compared with being in the lowest quartile, being in the highest quartile for meat intake was significantly related to a higher prevalence of rhinoconjunctivitis, and the positive linear trend was also significant in crude analysis. Adjustment for confounders under study did not materially alter the positive relationship: the adjusted OR between extreme quartiles was 1.71 (95% CI: 1.25-2.35, *P *for trend = 0.002). No measurable association was found between fish intake and rhinoconjunctivitis.

**Table 3:** Odds ratios (ORs) and 95% confidence intervals (CIs) for rhinoconjunctivitis by quartiles of intake of meat and fish in 1745 pregnant women, Kyushu Okinawa Maternal and Child Health Study, Japan

Variable	Quartile				
	
	1 (Lowest) (n = 436)	2 (n = 436)	3 (n = 436)	4 (Highest) (n = 437)	*P *for trend
Meat					
Intake, g/day*	35.1	54.0	70.2	95.6	
Prevalence, %^†^	20.6	26.2	26.2	30.7	
Crude OR (95% CI)	1.00	1.36 (0.99-1.87)	1.36 (0.99-1.87)	1.70 (1.25-2.32)	0.001
Adjusted OR (95% CI)^‡^	1.00	1.37 (0.99-1.89)	1.34 (0.97-1.85)	1.71 (1.25-2.35)	0.002
Fish					
Intake, g/day*	22.8	37.2	49.5	71.7	
Prevalence, %^†^	26.8	24.3	27.3	25.2	
Crude OR (95% CI)	1.00	0.88 (0.65-1.19)	1.02 (0.76-1.38)	0.92 (0.68-1.24)	0.83
Adjusted OR (95% CI)^‡^	1.00	0.82 (0.60-1.12)	0.98 (0.72-1.33)	0.87 (0.63-1.19)	0.63

* Values for intake are medians for adjusted energy intake using the residual method for each quartile.

^† ^Prevalence of rhinoconjunctivitis based on the International Study of Asthma and Allergies in Childhood for each quartile.

^‡ ^Adjustment for age; gestation; region of residence; number of older siblings; number of children; smoking; secondhand smoke exposure at home and at work; family history of asthma, atopic eczema, and allergic rhinitis; household income; education; and body mass index.

Results for intake of specific types of fatty acids and cholesterol are given in Table 4. Intake of total fat, saturated fatty acids, monounsaturated fatty acids, n-3 polyunsaturated fatty acids, α-linolenic acid, eicosapentaenoic acid, docosahexaenoic acid, n-6 polyunsaturated fatty acids, linoleic acid, arachidonic acid, and cholesterol and the ratio of n-3 to n-6 polyunsaturated fatty acid intake were not significantly related to the prevalence of rhinoconjunctivitis in the multivariate model.

**Table 4:** Odds ratios (ORs) and 95% confidence intervals (CIs) for rhinoconjunctivitis by quartiles of intake of specific fats in 1745 pregnant women, Kyushu Okinawa Maternal and Child Health Study, Japan

	Quartile				
	
Variable	1 (Lowest) (n = 436)	2 (n = 436)	3 (n = 436)	4 (Highest) (n = 437)	*P *for trend
Total fat					
Intake, g/day*	47.2	54.7	60.4	69.7	
Prevalence, %^†^	23.9	25.2	25.7	28.8	
Crude OR (95% CI)	1.00	1.08 (0.79-1.47)	1.10 (0.81-1.50)	1.29 (0.96-1.75)	0.10
Adjusted OR (95% CI)^‡^	1.00	1.09 (0.79-1.49)	1.11 (0.81-1.53)	1.31 (0.96-1.79)	0.09
Saturated fatty acids					
Intake, g/day*	12.6	15.5	17.6	21.2	
Prevalence, %^†^	25.7	25.2	23.6	29.1	
Crude OR (95% CI)	1.00	0.98 (0.72-1.32)	0.90 (0.66-1.22)	1.19 (0.88-1.60)	0.36
Adjusted OR (95% CI)^‡^	1.00	0.99 (0.72-1.35)	0.96 (0.70-1.32)	1.20 (0.88-1.63)	0.29
Monounsaturated fatty acids					
Intake, g/day*	15.7	18.9	21.2	25.0	
Prevalence, %^†^	24.3	25.0	25.9	28.4	
Crude OR (95% CI)	1.00	1.04 (0.76-1.41)	1.09 (0.80-1.48)	1.23 (0.91-1.67)	0.16
Adjusted OR (95% CI)^‡^	1.00	0.99 (0.72-1.36)	1.08 (0.79-1.48)	1.19 (0.87-1.63)	0.21
n-3 Polyunsaturated fatty acids					
Intake, g/day*	1.7	2.1	2.4	2.9	
Prevalence, %^†^	25.5	25.2	24.8	28.2	
Crude OR (95% CI)	1.00	0.99 (0.73-1.34)	0.96 (0.71-1.31)	1.15 (0.85-1.55)	0.42
Adjusted OR (95% CI)^‡^	1.00	0.92 (0.67-1.26)	0.90 (0.65-1.23)	1.07 (0.78-1.45)	0.72
α-Linolenic acid					
Intake, g/day*	1.2	1.5	1.8	2.2	
Prevalence, %^†^	25.0	23.2	28.2	27.2	
Crude OR (95% CI)	1.00	0.90 (0.66-1.23)	1.18 (0.87-1.59)	1.12 (0.83-1.52)	0.21
Adjusted OR (95% CI)^‡^	1.00	0.88 (0.64-1.21)	1.18 (0.86-1.60)	1.04 (0.76-1.42)	0.43
Eicosapentaenoic acid					
Intake, g/day*	0.07	0.12	0.17	0.29	
Prevalence, %^†^	25.7	25.2	27.1	25.6	
Crude OR (95% CI)	1.00	0.98 (0.72-1.32)	1.07 (0.79-1.45)	1.00 (0.74-1.35)	0.86
Adjusted OR (95% CI)^‡^	1.00	0.91 (0.67-1.25)	1.00 (0.73-1.37)	0.93 (0.68-1.28)	0.81
Docosahexaenoic acid					
Intake, g/day*	0.14	0.22	0.30	0.46	
Prevalence, %^†^	25.2	25.2	29.4	23.8	
Crude OR (95% CI)	1.00	1.00 (0.74-1.36)	1.23 (0.91-1.66)	0.93 (0.68-1.26)	0.98
Adjusted OR (95% CI)^‡^	1.00	0.93 (0.68-1.27)	1.13 (0.83-1.54)	0.88 (0.64-1.21)	0.73
n-6 Polyunsaturated fatty acids					
Intake, g/day*	8.5	10.2	11.5	13.5	
Prevalence, %^†^	24.3	23.2	30.1	26.1	
Crude OR (95% CI)	1.00	0.94 (0.69-1.28)	1.34 (0.99-1.81)	1.10 (0.81-1.49)	0.19
Adjusted OR (95% CI)^‡^	1.00	0.91 (0.66-1.26)	1.34 (0.98-1.83)	1.05 (0.77-1.45)	0.29
Linoleic acid					
Intake, g/day*	8.3	10.0	11.3	13.1	
Prevalence, %^†^	24.3	23.6	29.1	26.5	
Crude OR (95% CI)	1.00	0.96 (0.71-1.31)	1.28 (0.95-1.73)	1.13 (0.83-1.53)	0.19
Adjusted OR (95% CI)^‡^	1.00	0.94 (0.68-1.29)	1.27 (0.93-1.73)	1.08 (0.79-1.48)	0.30
Arachidonic acid					
Intake, g/day*	0.09	0.12	0.14	0.18	
Prevalence, %^†^	25.0	24.1	28.4	26.1	
Crude OR (95% CI)	1.00	0.95 (0.70-1.30)	1.19 (0.88-1.61)	1.06 (0.78-1.44)	0.42
Adjusted OR (95% CI)^‡^	1.00	0.90 (0.65-1.23)	1.20 (0.88-1.63)	1.05 (0.77-1.43)	0.39
n-3/n-6 Polyunsaturated fatty acid ratio					
Intake*	0.17	0.19	0.21	0.25	
Prevalence, %^†^	24.8	26.8	27.8	24.3	
Crude OR (95% CI)	1.00	1.11 (0.82-1.51)	1.17 (0.86-1.58)	0.97 (0.71-1.32)	0.95
Adjusted OR (95% CI)^‡^	1.00	1.07 (0.78-1.46)	1.07 (0.78-1.46)	0.92 (0.67-1.26)	0.61
Cholesterol					
Intake, mg/day*	182.0	244.5	308.0	402.7	
Prevalence, %^†^	26.4	22.5	28.0	26.8	
Crude OR (95% CI)	1.00	0.81 (0.59-1.10)	1.09 (0.81-1.46)	1.02 (0.76-1.38)	0.48
Adjusted OR (95% CI)^‡^	1.00	0.82 (0.60-1.13)	1.10 (0.81-1.49)	1.05 (0.77-1.42)	0.41

* Values for intake are medians for adjusted energy intake using the residual method for each quartile.

^† ^Prevalence of rhinoconjunctivitis based on the International Study of Asthma and Allergies in Childhood for each quartile.

^‡ ^Adjustment for age; gestation; region of residence; number of older siblings; number of children; smoking; secondhand smoke exposure at home and at work; family history of asthma, atopic eczema, and allergic rhinitis; household income; education; and body mass index.

## Discussion

The current cross-sectional study of pregnant Japanese women revealed that higher meat intake was independently associated with a higher prevalence of rhinoconjunctivitis. There were no significant associations between intake of total fat, saturated fatty acids, monounsaturated fatty acids, n-3 polyunsaturated fatty acids, n-6 polyunsaturated fatty acids, or cholesterol or the ratio of n-3 to n-6 polyunsaturated fatty acid intake and the prevalence of rhinoconjunctivitis.

Our results are not consistent with the hypothesis that decreased intake of n-3 polyunsaturated fatty acids and increased intake of n-6 polyunsaturated fatty acids might have contributed to the recent increase in the prevalence of allergic disorders [4], a hypothesis that is supported by several other studies. In two cross-sectional studies of German children, a significant positive association was observed between margarine intake and allergic rhinitis [5,6]. A significant inverse association between fish intake and allergic rhinitis was shown in two cohort studies of children in Norway [9] and Sweden [10]. A recent prebirth cohort study in Finland found that higher maternal α-linolenic acid intake during pregnancy was significantly related to a reduced risk of allergic rhinitis in children by 5 years of age, while a significant positive association was found between the ratio of n-6 to n-3 polyunsaturated fatty acid intake during pregnancy and childhood allergic rhinitis [12]. On the other hand, there was no relationship between fish intake and allergic rhinitis in a cross-sectional study of Italian children [8]. In a Finnish study, the differences in consumption of margarine and fish were not significant between children with allergic rhinitis and those without [7]. With regard to studies in adults, a cross-sectional study of German adults showed significant positive associations between margarine intake and the ratio of n-6 to n-3 polyunsaturated fatty acid intake and hay fever in men, but not in women [14]. In a case-control study of German adults, higher eicosapentaenoic acid intake was significantly associated with a reduced risk of hay fever [13]. In young adult Japanese women, a significant inverse relationship was observed between intake of marine origin n-3 polyunsaturated fatty acids [16] or seafood [17] and allergic rhinitis. A significant inverse relationship between α-linolenic acid intake and the prevalence of allergic rhinitis was found in German adults [15]. The discrepancies among studies may be explained, at least in part, by differences in the study populations and designs, dietary assessment methods, definitions of allergic rhinitis, and confounders.

Japanese people eat considerably more fish than do people in Western countries. The mean values for daily intake of eicosapentaenoic and docosahexaenoic acids in this population were 0.17 g and 0.29 g, respectively. In many parts of Europe, the daily combined intake of eicosapentaenoic acid and docosahexaenoic acid by adults is < 100 mg [24]. A beneficial association between n-3 polyunsaturated fatty acid intake and rhinoconjunctivitis might be detected among populations whose fish intake is low. Alternatively, unknown active agents in fish might have interfered with the beneficial effect of marine-origin n-3 polyunsaturated fatty acids in rhinoconjunctivitis. For example, methylmercury and dioxins are accumulated in fish and shellfish through the marine food web. A significant correlation between fish consumption and hair mercury levels was found, and hair mercury levels were much higher in Japanese women residing in Canada than in Canadian women [25].

To our knowledge, this study is the first to show a significant positive association between meat intake and the prevalence of rhinoconjunctivitis. The current findings regarding meat intake are in partial agreement with those of a previously cited Japanese study of female university students that showed a significant positive relationship between meat intake and the prevalence of wheeze, but not between meat intake and pollen allergy [17]. The present findings are also partially consistent with those of a prebirth cohort study in Japan that reported that higher maternal meat intake during pregnancy was significantly associated with an increased risk of suspected atopic eczema, which was based on a physician's diagnosis of atopic eczema or possible atopic eczema, in infants aged 3-4 months [26], although such intake was not related to the risk of wheeze or eczema based on the ISAAC criteria in the children aged 16-24 months [27]. In reports using baseline data from the prebirth cohort study in Japan, there were no associations between meat intake and the prevalence of asthma, atopic eczema, or allergic rhinitis in pregnant women [16,28,29]. Several studies showed no relationships between meat intake and wheeze and/or asthma in children [30-36] and adults [37]. Furthermore, a significant inverse association was found between red meat intake and doctor-diagnosed asthma in a cross-sectional study of young Australian adults [38]. These observations are at variance with the current results. The positive association between meat intake and rhinoconjunctivitis in this study is not ascribed to saturated fatty acid intake because saturated fatty acid intake was not related to rhinoconjunctivitis. The other constituents in meat may affect the development of rhinoconjunctivitis. Cooking meat at high temperatures results in the formation of carcinogenic compounds, such as heterocyclic amines and polycyclic aromatic hydrocarbons [39]. Also, meat intake results in exposures to *N*-nitroso compounds, which can form endogenously [40] and exogenously in nitrite-preserved meats [41]. Our results regarding meat intake might be ascribed to these possible carcinogens; however, there is no epidemiological evidence for relationships between these carcinogens and allergic disorders. Alternatively, meat intake might be a marker of a dietary pattern that is related to rhinoconjunctivitis. Or, some unknown non-dietary factors related to meat intake may have confounded the observed association. Higher meat intake is likely to be linked to a westernized lifestyle which may be related to rhinoconjunctivitis among Japanese.

This study has methodological advantages. Study subjects were homogeneous in that they were all pregnant. We took into consideration extensive information on potential confounders. Adjustment was not made for external factors such as aeroallergens and air pollution, however. The definition of rhinoconjunctivitis was based on the questions of the ISAAC, although validation tests of these questions have not been performed for young Japanese adults.

There are methodological limitations, however. Our DHQ could only approximate consumption. The validity of our DHQ seems reasonable, as described above, although the correlation coefficient for polyunsaturated fatty acids was 0.37 [19]. The possibility of exposure misclassification would bias the estimates of the associations toward the null. Our DHQ was designed to assess dietary intake for one month prior to completing the questionnaire. Information on dietary supplements that included eicosapentaenoic acid and docosahexaenoic acid was not available; however, it might not be necessary to take such supplements into consideration among our study subjects because pregnant Japanese women are generally not advised to take such supplements. Only a small number of our participants used vitamin C (4.6%), multivitamin (5.5%), and calcium (5.6%) supplements at least once a week, although 26.2% used vitamin B complex at least once a week. Substantial changes in diet in the previous month were experienced by 502 pregnant women because of nausea gravidarum (473 women), maternal and fetal health (27 women), and other reasons (2 women). The results of a sensitivity analysis excluding these 502 women were similar to those in the overall analysis: the adjusted OR for rhinoconjunctivitis in the highest quartile of meat intake was 1.47 (95% CI: 1.01-2.15, *P *for trend = 0.04).

The participation rate could not be calculated because the exact number of eligible pregnant women who were provided with a set of leaflets explaining the KOMCHS, an application form, and a self-addressed and stamped return envelope by the 423 collaborating obstetric hospitals was not available. We were not able to analyze the differences between participants and non-participants, because information on personal characteristics such as age, socioeconomic status, and history of allergic disorders was not available for non-participants. It is likely, however, that our subjects were not a representative sample of Japanese women in the general population. In fact, educational levels in the present study population were higher than in the general population. According to the 2000 population census of Japan, the proportions of women aged 30 to 34 years in Fukuoka Prefecture with < 13, 13-14, ≥ 15, and an unknown number of years of education were 52.0%, 31.5%, 11.8%, and 4.8%, respectively [42]. The corresponding figures for the current study were 24.5%, 33.1%, 42.4%, and 0.0%, respectively. The present population might therefore have had greater awareness of health topics than did the general population.

The cross-sectional nature of the study did not permit the assessment of causality because of the uncertain temporality of the association. Therefore, the findings from the present cross-sectional study do not necessarily indicate a causal relationship between meat intake and rhinoconjunctivitis.

The interface between allergy/immunology and pregnancy should be discussed, as it may have an influence on the association of interest. It has been suggested that pregnancy involves a shift to the Th2 side of the immune response [43] although Chaouat et al. have pointed out the importance of the role of natural killer cells and IL-12, IL-15, and IL-18 tripods in successful or failed pregnancies in humans beyond the Th1/Th2 paradigm [44]. The hormonal changes in pregnancy are often invoked to explain the apparent association between rhinitis symptoms and pregnancy. However, rhinitis ascribed solely to pregnancy may not be a distinct entity because most pregnant women do not have significant nasal symptoms [43].

## Conclusion

The current results suggest that higher meat intake may be associated with an increased prevalence of rhinoconjunctivitis in young adult Japanese women. This finding warrants further epidemiological investigation of diet and allergic rhinitis, especially in prospective cohort studies.

## Abbreviations

CI: Confidence interval; DHQ: Diet history questionnaire; ISAAC: International Study of Asthma and Allergies in Childhood; KOMCHS: Kyushu Okinawa Maternal and Child Health Study; OR: Odds ratio.

## Competing interests

The authors declare that they have no competing interests.

## Authors' contributions

YM, KT, and MA contributed to the study concept and design and the acquisition of data. HO and SS were responsible for the estimation of dietary factors. YM was responsible for the analysis and interpretation of data and the drafting of the manuscript. All authors read and approved the final manuscript.
